# Using Statistical Measures and Density Maps Generated From Chest Computed Tomography Scans to Identify and Monitor COVID-19 Cases in Radiation Oncology Rapidly

**DOI:** 10.7759/cureus.17432

**Published:** 2021-08-25

**Authors:** Marie-Hélène Tomé, Megi Gjini, Shaoyu Zhu, Rafi Kabarriti, Chandan Guha, Madhur K Garg, Wolfgang A Tomé, N. Patrik Brodin

**Affiliations:** 1 Institute for Onco-Physics, Albert Einstein College of Medicine, Bronx, USA; 2 Radiation Oncology, Montefiore Medical Center and Albert Einstein College of Medicine, Bronx, USA

**Keywords:** covid-19, chest ct, screening tools, density maps, decision-support

## Abstract

Objectives

This study aimed to evaluate quantitative and qualitative screening measures for anomalous computed tomography (CT) scans in cancer patients with potential coronavirus disease 2019 (COVID-19) as an automated detection tool in a radiation oncology treatment setting.

Methods

We identified a non-COVID-19 cohort and patients with suspected COVID-19 with chest CT scans from February 1, 2020 to June 30, 2020. Lungs were segmented, and a mean normal Hounsfield Unit (HU) histogram was generated for the non-COVID-19 CT scans; these were used to define thresholds for designating the COVID-19-suspected histograms as normal or abnormal. Statistical measures were computed and compared to the threshold levels, and density maps were generated to examine the difference between lungs with and without COVID-19 qualitatively.

Results

The non-COVID-19 cohort consisted of 70 patients with 70 CT scans, and the cohort of suspected COVID-19 patients consisted of 59 patients with 80 CT scans. Sixty-two patients were positive for COVID-19. The mean HUs and skewness of the intensity histogram discriminated between COVID-19 positive and negative cases, with an area under the curve of 0.948 for positive and 0.944 for negative cases. Skewness correctly identified 57 of 62 positive cases, whereas mean HUs correctly identified 17 of 18 negative cases. Density maps allowed for visualization of the temporal evolution of COVID-19 disease.

Conclusions

The statistical measures and density maps evaluated here could be employed in an automated screening algorithm for COVID-19 infection. The accuracy is high enough for a simple and rapid screening tool for early identification of suspected infection in patients treated with chemotherapy and radiation therapy already receiving CT scans as part of clinical care. This screening tool could also identify other infections that present critical risks for patients undergoing chemotherapy and radiation therapy, such as pneumonitis.

## Introduction

As of January 5, 2021, there have been over 86 million cases of coronavirus disease 2019 (COVID-19), with more than 1.8 million deaths, worldwide [1]. COVID-19 is a disease secondary to infection from severe acute respiratory syndrome coronavirus 2 that can cause pneumonia and lung damage in symptomatic cases. The effect of COVID-19 on the lungs is visible in chest computed tomography (CT) scans. Since radiation oncology patients receive numerous CT scans for their diagnostic work-up, treatment planning, and tumor positioning during treatment, these scans could be leveraged to implement an automated screening tool. Patients receiving cancer treatment are more vulnerable to COVID-19 and have a higher incidence of severe cases and mortality [2,3]. Respiratory illnesses, hypertension, and diabetes have also been identified as risk factors for poor COVID-19 outcomes [4].

Initial signs of COVID-19 in the lungs might not be detected by chest radiography as they can develop in areas difficult to visualize on radiographs [5]. CT scans are, therefore, superior to chest radiography in detecting early signs of COVID-19. Increased lung density due to COVID-19 is typically found bilaterally and in the peripheral regions of the lungs [6]. CT scans were part of primary diagnoses for COVID-19 before polymerase chain reaction (PCR) swab tests became readily available. Studies on the diagnostic potential of CT scans were not typically able to distinguish between COVID-19 pneumonia and influenza-related pneumonia since both cause an increase in lung density [7]. Therefore, CT scans are no longer the first-line test for patients with suspected COVID-19.

However, the high sensitivity of CT scans can be leveraged as a screening tool, especially for patients with lung cancer, esophageal cancer, or other pulmonary indications who undergo routine CT scans as a part of their diagnostic work-up and treatment. With PCR tests readily available in most hospital systems, the high sensitivity of a CT-based screening tool can be used to identify suspected cases early and determine diagnosis using PCR testing. Furthermore, the ability to automate the rapid detection of any infection leading to respiratory disease in cancer patients would allow early intervention in this highly vulnerable patient population.

Here, we propose using statistical measures derived from Hounsfield Unit (HU) intensity volume histograms and lung density maps as a potential rapid screening tool for COVID-19 in radiation oncology patients already receiving chest CT scans as part of their clinical workflow.

## Materials and methods

Selection of patients and chest CT scans

Albert Einstein College of Medicine Institutional Review Board (IRB) issued approval for this study (approval number 2016-6665; approval date 03/01/2021) by expedited review under 45 CFR 46.110 and 21 CFR 56.110. Based on the IRB-approved study protocol, we identified patients with chest CT examinations. One patient cohort consisted of patients receiving CT scans between January 1, 2019 and August 31, 2019, before the COVID-19 pandemic in the United States. Here, we selected an equal number of patients with and without contrast-enhanced CT scans as the control non-COVID-19 cohort. The other cohort consisted of patients with suspected COVID-19 with CT scans acquired between February 1, 2020 and June 30, 2020, and any CT scans for those patients acquired in the first eight months of 2019 as internal controls. Following patient selection, the CT scans were exported to MIM software (MIM Software Inc., Cleveland, OH) for lung segmentation.

Lung segmentation and data processing

The chest CT scans consisted of 5-mm slice thickness images with 512 x 512 pixels, with each scan consisting of approximately 50 to 70 slices covering the entire chest. The lungs (right and left lung together) were then segmented in MIM using a semi-automatic region-growing algorithm and manually adjusted as necessary to include all lung tissue. If there existed known non-COVID-19 pathologies such as a lung tumor, this was not included in the lung contours. Once completed, the CT scan data and lung segmentation were anonymized and exported to MATLAB (MathWorks, Natick, MA) for data processing and analysis, masking the CT data to only include the segmented lung contours as a three-dimensional (3D) image structure.

Data analysis

The CT data for the cohort of control non-COVID-19 scans were used to generate a normal mean histogram of CT HUs. Before this, any HUs above 200 were excluded to avoid the inclusion of higher density anatomy at the fringes of the lung contours. To make the histograms of lung HUs comparable between different CT scans and patients, we normalized the histograms to 100,000 data points binned into 1,200 bins ranging from -1000 to 200 HUs. The normal mean histogram was then generated by adding all of the normalized histograms of non-COVID-19 CT scans and dividing by the number of scans included.

To quantitatively differentiate between control scans and scans showing COVID-19, we included simple statistical measures such as the mean, median, and skewness of the lung HU histogram and the proportion of data points falling above the mean. We also included statistical distance measures, such as the Kolmogorov-Smirnov (KS) distance, the Earth Mover’s Distance (EMD), and the Cramér-von-Mises (CvM) distance as further quantitative measures to differentiate the CT scans. These statistical distance measures account for all information in a lung HU histogram and compare it to the mean normal histogram.

The data from the normal mean histogram of the non-COVID-19 cohort were used to define thresholds for all the study metrics to designate scans as either normal or abnormal. A scan was classified as abnormal for a given study metric if it exceeded the 99% confidence limit of the mean normal histogram. Subsequently, all suspected COVID-19 CT scans were normalized and processed in the same way as described above, and the statistical measures for each scan were computed and compared to the established threshold levels.

**Figure 1:**
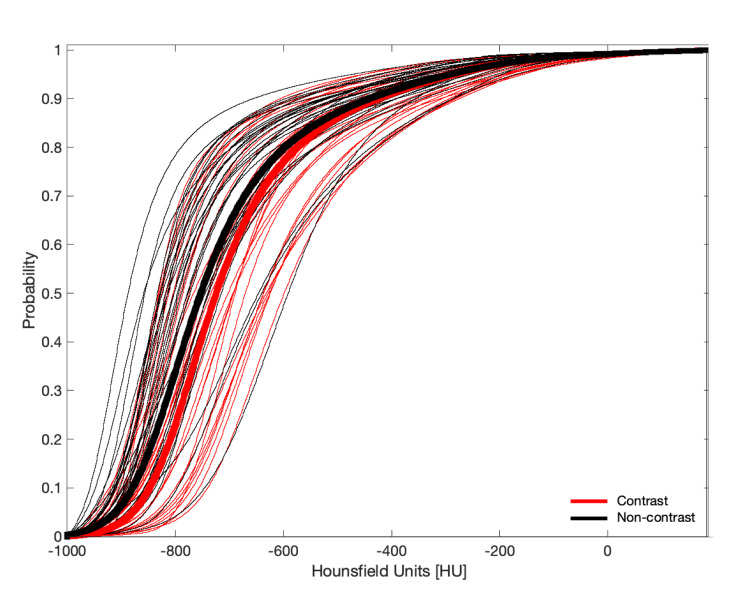
Average normal cumulative distribution functions of the non-COVID-19 cohort Bold-red curve - for patients who received contrast-enhanced CT scans; Bold-black curve - for patients who received noncontrast CT scans; Thin red and black curves - corresponding individual cumulative distribution functions of the non-COVID-19 patients COVID-19 - Coronavirus disease 2019; CT - Computed tomography.

We found that the mean normal cumulative lung histograms generated from CT scans of patients that received contrast compared to noncontrast scans were not meaningfully different (Figure 1). Therefore, the two groups were combined in this analysis. As a sensitivity analysis was performed, separate analyses were conducted using thresholds derived separately for contrast and noncontrast scans. A further sensitivity analysis was performed by stratifying patients by age.

Visual density maps were constructed from the lung contour data using Tool for OPerations on Catalogues And Tables (TOPCAT) version 4.8 (TOPCAT, Bristol, England) freeware astrophysics graphing tool. For noncontrast CT scans, the threshold below which all HU values were excluded in the maps was -150 HU. For contrast scans, this threshold was -100 HU. The lungs were compressed in two dimensions along the cranial-caudal axis, plotting the count of voxels above the threshold along this axis in the transverse plane. These density maps were then visualized on a logarithmic scale.

Statistical analysis

The baseline for COVID-19 status and disease severity at the time of CT scan was established by retrospectively examining the patient’s medical records and PCR test results. This was performed by investigators blinded to any CT scan data. Cases were classified as either COVID-19-negative or positive at the time of CT scan, and positive cases classified as asymptomatic, mild, moderate, or severe disease. To classify the performance of the statistical measures, we computed the sensitivity, specificity, negative predictive value, positive predictive value, and accuracy at the given threshold level. The performance of each metric was determined by plotting the receiver operating characteristics (ROC) curve and calculating the accompanying area under the curve (AUC).

## Results

Seventy patients with 70 chest CT scans were identified for the non-COVID-19 cohort, and 59 patients with 80 chest CT scans were identified for the cohort of suspected COVID-19 cases; patient characteristics are presented in Table 1. Both patient cohorts spanned a broad age range with similar age distribution. The majority of suspected COVID-19 CT scans were acquired with contrast enhancement. Mild and moderate COVID-19 cases comprised most of the suspected COVID-19 cohort, with approximately one-third of the cases having moderate or severe disease. Within the suspected COVID-19 cohort, 18 of the suspected cases (22.5%) were negative at the time of CT scan.

**Table 1:** Patient and CT scan characteristics. CT - Computed tomography; COVID-19 - Coronavirus disease 2019; NA - Not applicable; SD - Standard deviation.

	Non-COVID-19 Cohort	Suspected COVID-19 Cohort
	(N = 70 scans in 70 patients)	(N = 80 scans in 59 patients)
Sex, n (%)		
Male	30 (43%)	44 (45%)
Female	40 (57%)	36 (55%)
Age, years		
Mean (± SD)	58 ± 17	62 ± 16
Range	11 – 90	21 – 88
Use of contrast, n (%)		
Contrast	35 (50%)	55 (69%)
Noncontrast	35 (50%)	25 (31%)
COVID-19 case severity at time of CT scan		
Negative	70 (100%)	18 (22.5%)
Asymptomatic	NA	6 (7.5%)
Mild	NA	30 (37.5%)
Moderate	NA	15 (19%)
Severe	NA	11 (14%)

**Figure 2:**
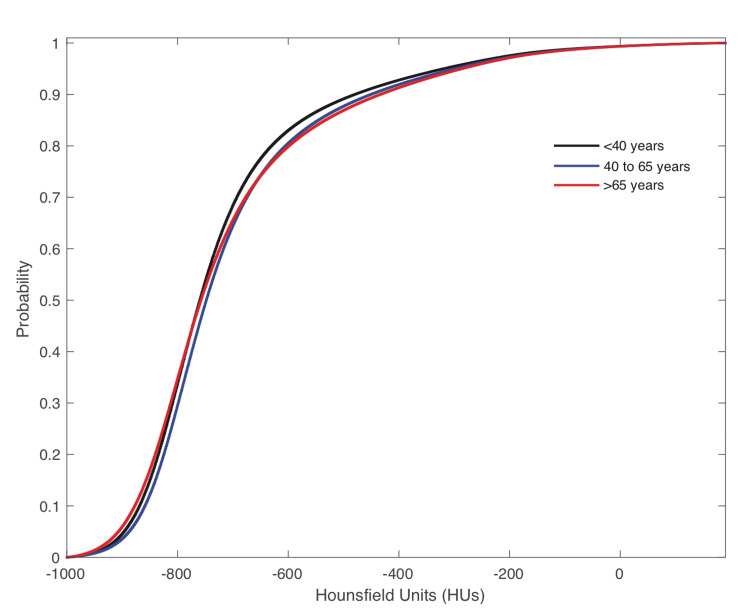
Average normal cumulative distribution functions of the non-COVID-19 cohort for patients stratified by age group as <40 years, 40 to 65 years, and >65 years. COVID-19 - Coronavirus disease 2019

Figure 2 shows only minor variations in the cumulative distribution of HUs between scans from patients in different age groups, and as such, we did not stratify the analysis based on age.

**Figure 3:**
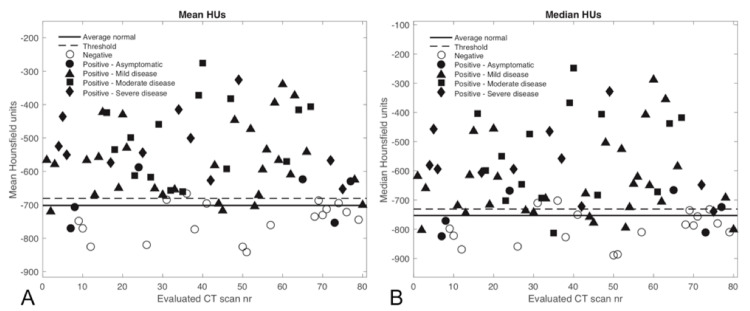
The considered statistical measures are shown for each evaluated CT scan along with the threshold levels determined from the mean normal histogram and the value from the average normal histogram for A) Mean HUs and B) Median HUs. Values above the threshold are considered abnormal. CT - Computed tomography; HU - Hounsfield units; nr - Number.

**Figure 4:**
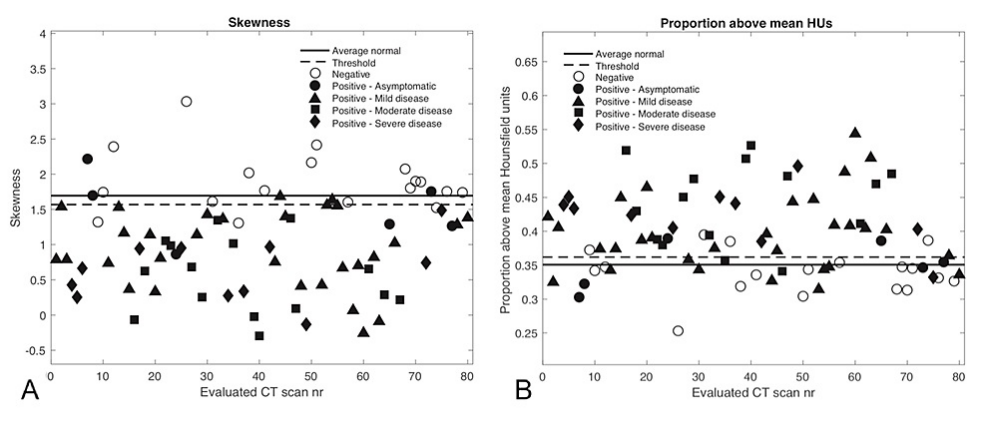
The considered statistical measures are shown for each evaluated CT scan along with the threshold levels determined from the mean normal histogram and the value from the average normal histogram for A) Skewness and B) Proportion above mean HUs. For the skewness (A), values below the threshold value are considered abnormal, and for (B) values above the threshold are considered abnormal. CT - Computed tomography; HU - Hounsfield units; nr - Number.

**Figure 5:**
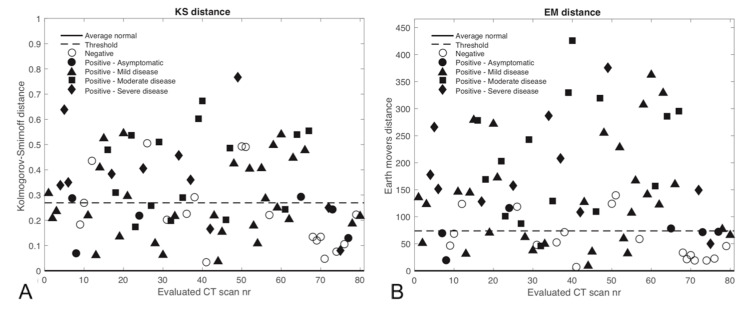
The considered statistical measures are shown for each evaluated CT scan along with the threshold levels determined from the mean normal histogram and the value from the average normal histogram for A) KS distance and B) EM distance. Values above the threshold are considered abnormal. CT - Computed tomography; HU - Hounsfield units; nr - Number; EM - Earth Mover’s; KS - Kolmogorov-Smirnov.

The statistical measures and classification of all 80 evaluated CT scans are shown in Figures 3-5. Since the CvM distance characterized cases similar to the EMD, it was not evaluated separately in a more limited range. Most of the negative scans are correctly classified as normal according to the various threshold levels depicted, except for the KS distance, which did not work well for classifying cases. 

The mean HU and skewness metrics showed good separation between positive and negative COVID-19 cases at the given threshold levels. Positive COVID-19 CT scans incorrectly classified as normal corresponded with asymptomatic or mild disease, whereas CTs corresponding to moderate or severe disease were classified as abnormal. The statistical measures could not distinguish well between moderate and severe cases because there was typically widespread pneumonia with a considerable increase in lung HU numbers in most of these instances. The threshold levels shown represent the 99% confidence limits from the average normal scan described in the methods and appear to be well suited for distinguishing positive from negative cases.

Table 2 shows the corresponding measures of classification performance. Skewness and mean HUs had the best performance for classifying negative or positive cases, with an accuracy of 0.900 and 0.888, respectively. Skewness has the best sensitivity at 0.919 with 57 of 62 true positives, while mean HUs had the best specificity at 0.944 with only one false-negative classification. The EMD showed decent classification performance, but it was not on par with the other statistical measures, and the KS distance was, again, not a good classification metric for this setting.

**Table 2:** Performance of considered statistical measures in classifying COVID-19 cases. COVID-19 - Coronavirus disease 2019; EMD - Earth Mover’s distance; KS - Kolmogorov-Smirnov; HUs - Hounsfield units.

	Mean HUs	Median HUs	Proportion above Mean HUs	Skewness	EMD	KS distance
True positive	54/62	49/62	46/62	57/62	45/62	33/62
True negatives	17/18	16/18	14/18	15/18	14/18	13/18
Sensitivity	0.871	0.790	0.742	0.919	0.726	0.532
Specificity	0.944	0.889	0.778	0.833	0.778	0.722
Positive predictive value	0.982	0.961	0.920	0.950	0.918	0.868
Negative predictive value	0.680	0.552	0.467	0.750	0.452	0.310
Accuracy	0.888	0.813	0.750	0.900	0.738	0.575

**Figure 6:**
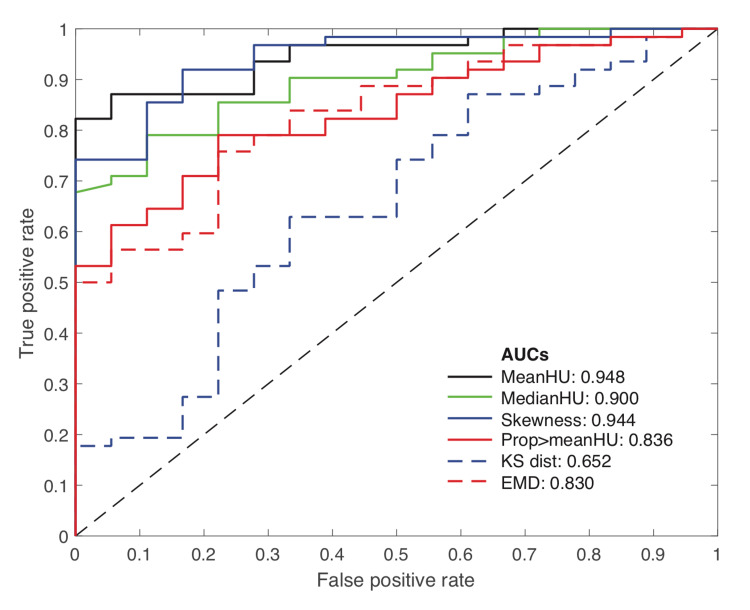
Receiver Operator Characteristics (ROC) curves for the considered statistical measures and corresponding area under the curve measurements. AUCs - Areas under the curve; HU - Hounsfield units; KS dist - Kolmogorov-Smirnov distance; EMD - Earth Mover’s Distance.

The ROC curves for each of the studied statistical measures are presented in Figure 6, which compares their performance for classifying positive or negative cases independent of the specific threshold level chosen. The ROC curves show that mean HUs and skewness have the best classification performance with AUC values of 0.948 and 0.944, respectively. While median HUs also showed good classification performance with an AUC of 0.900, the other metrics did not perform as well, corroborating the results presented in Table 2 above.

**Figure 7:**
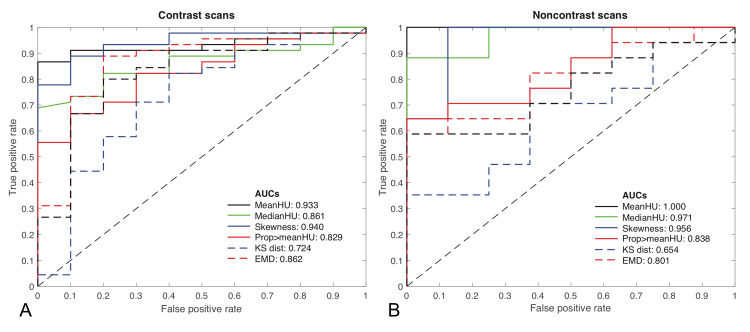
Receiver Operator Characteristics (ROC) curves for the considered statistical measures and corresponding area under the curve measurements stratified by contrast-enhanced CT scans (A) and noncontrast CT scans (B). AUCs - Areas under the curve; CT - Computed tomography; HU - Hounsfield units; KS - Kolmogorov-Smirnov; EMD - Earth Mover’s Distance.

Figure 7 shows the classification performance ROC curves if stratifying the analysis between contrast (Figure 7A) and noncontrast (Figure 7B) CT scans. The AUC results are similar to Figure 6, where all scans are assessed together, with some potential improvement for noncontrast scans albeit with a small sample size of scans (n=25) in this group. 

**Figure 8:**
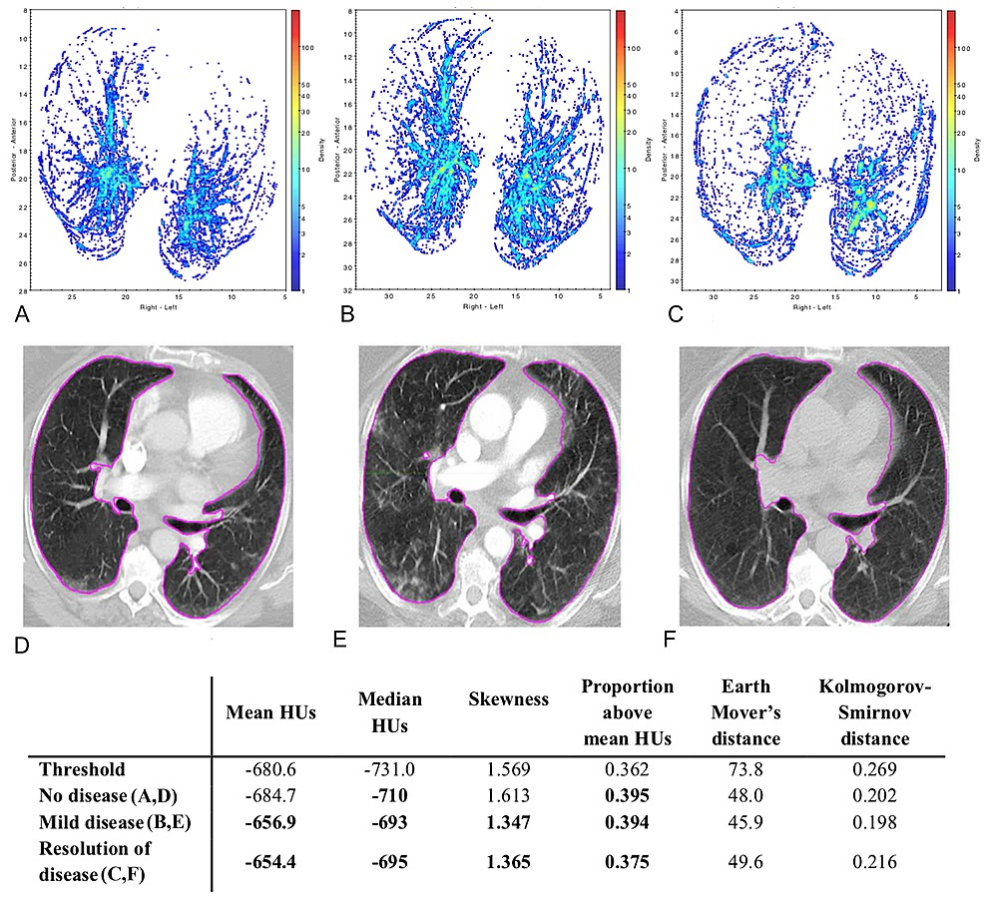
Temporal evolution of COVID-19 for a patient over a 9-month period (A, D) - no disease in 2019; (B, E) - mild disease in April 2020; (C, F) - resolution of disease in June 2020 A bolded entry in the table signifies that the statistical measure classified the scan as abnormal based on the given threshold. COVID-19 - Coronavirus disease 2019; HU - Hounsfield units.

**Figure 9:**
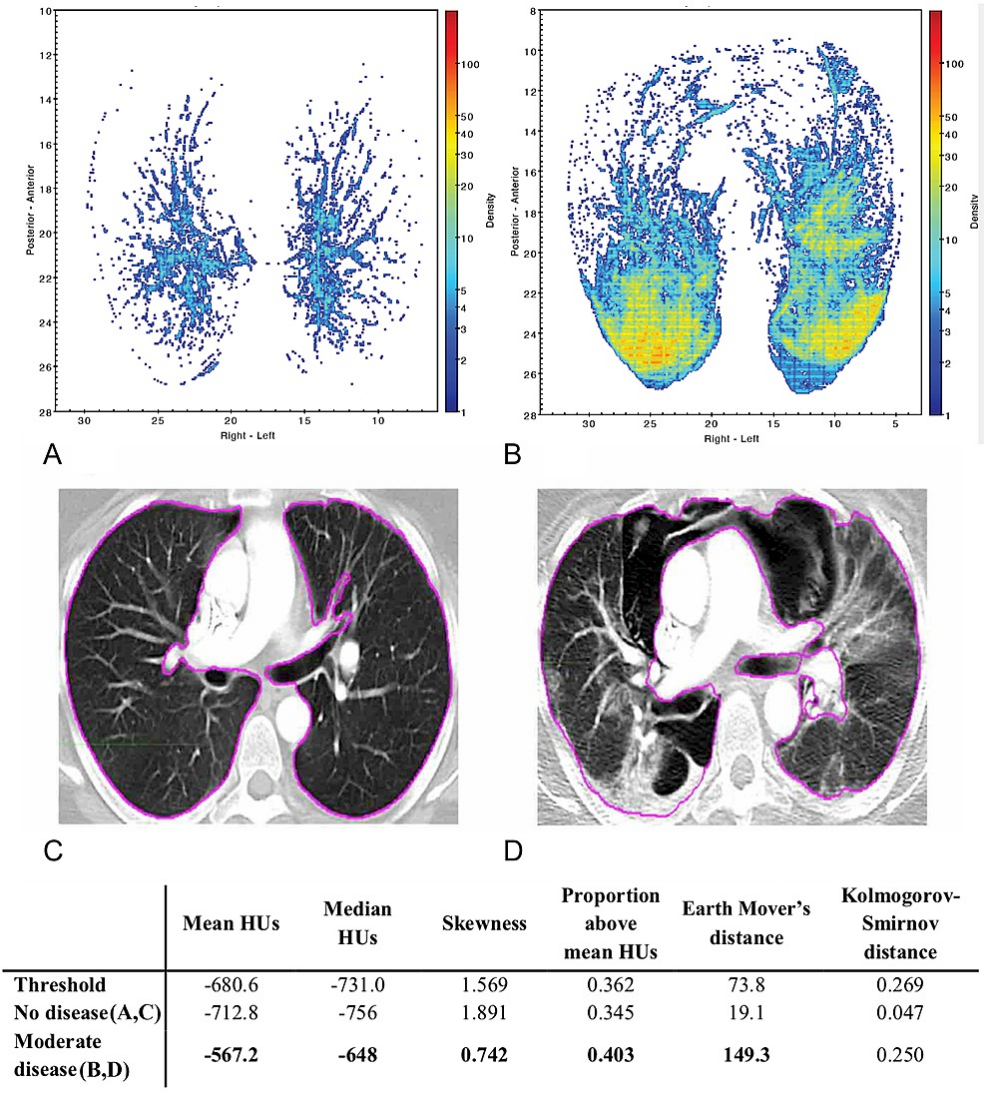
Comparison of scans pre- and post-COVID-19 infection in a patient (A, C) - no disease in March 2020; (B, D) - a moderate disease in April 2020 A bolded entry in the table signifies that the statistical measure classified the scan as abnormal based on the given threshold. COVID-19 - Coronavirus disease 2019; HU - Hounsfield units.

Figures 8, 9 present density maps for two different patients, showing the increase in lung tissue density associated with COVID-19 and how the density maps can illustrate signs of more severe disease. Using these maps, the information contained in the 3D CT scan is compressed into a two-dimensional map that allows for visualization of the COVID-19 disease burden across the entire lung in one snapshot. The density maps showed that the posterior of the lungs contained the greatest disease burden, especially for moderate and severe diseases. Comparing the density maps with the statistical measures shows the agreement between the visual qualitative interpretation of these maps with the quantitative assessment of whether to classify a scan as abnormal. The density maps can be particularly useful in tracking the progression or resolution of disease burden over time, as shown by the examples in Figures 8, 9.

## Discussion

This study focused on developing a quick and straightforward potential screening tool for COVID-19 in vulnerable patients treated in a radiation oncology setting. We found that simple statistical measures of mean HUs and skewness of the intensity histogram of chest CT scans performed well in discriminating between positive and negative cases of COVID-19. Skewness had the highest sensitivity of 0.919 in the testing cohort, while mean HUs had the highest specificity of 0.944, as they offer some complimentary classification performance. This screening tool could be implemented as a quick semi-automated test applicable to any patient for whom a chest CT scan is acquired. As a correlate, the density maps generated from the segmented lungs in the CT scan act as visual aids to further characterize COVID-19 disease progression and resolution. CT scans are sometimes difficult to use for characterizing lesion growth due to the extent of disease consolidation, whereas density maps may illustrate density differences between seemingly similar regions on a CT scan more easily. More severe cases typically show consolidation at the posterior of the lungs and are suggestive of inflammation of the vasculature in milder cases [7,8]. Further corroborating the agreement between such findings and density maps as presented here could provide an interesting avenue towards improved disease classification and perhaps monitoring efficacy of various COVID-19 treatments using the suggested screening tool.

A decline in pulmonary function and changes in lung morphology are associated with natural aging [9]. There were no appreciable differences in the mean normal cumulative distribution of HUs from different age groups for our patient cohort when stratified in our non-COVID-19 cohort (Figure 2). However, in a more extensive study, we expect small differences in the mean normal cumulative distribution functions of different age groups could require stratification by age to improve classification performance further. Similarly, a larger cohort study could stratify CT scans using contrast compared with noncontrast, which had some indication of potentially improving the classification performance further but would require a larger sample size to answer definitively.

**Figure 10:**
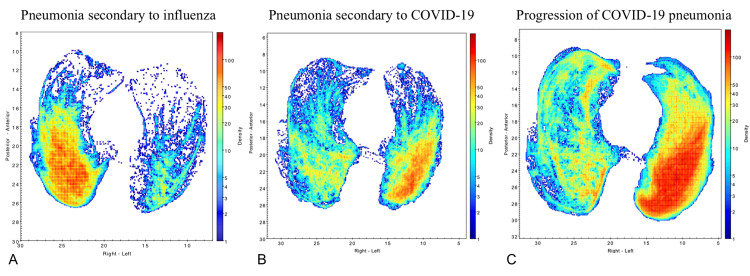
Lung CT scan density maps for a patient with secondary pneumonia attributable to influenza in December 2019 who developed COVID-19 pneumonia in April 2020 (A) Pneumonia secondary to influenza; (B) Pneumonia secondary to COVID-19; (C) Progression of COVID-19 pneumonia COVID-19 - Coronavirus disease 2019; CT - Computed tomography.

The high accuracy of the presented point measures would allow CT scans to be used as rapid screening tools, especially at the height of an outbreak when testing resources and labs are overburdened. A recent study supports this, with rapid diagnosis of COVID-19 from CT scans using convolutional neural networks showing promising results that align with our findings, especially when combined with clinical symptoms and laboratory serology [10]. The characteristics of COVID-19 on CT scans present a possibility to create more precise screening algorithms rather than just the simple metrics presented here. To this end, there have been virtual imaging trials conducted to exploit differences between COVID-19 and other respiratory illnesses to train artificial intelligence algorithms to distinguish COVID-19 specifically, based on CT scans [11]. One patient in the test cohort had secondary pneumonia attributable to influenza in December 2019 and then developed COVID-19 pneumonia in April 2020. The density maps from the CT scans for this patient showed some differences between COVID-19 and influenza-related pneumonia, but both were picked up by the classification algorithm (Figure 10).

## Conclusions

The statistical point measures evaluated in this study showed good classification performance and could be implemented as part of an automated screening tool algorithm for COVID-19, especially during the height of a pandemic. With this type of methodology, an automated algorithm could be trained on several virtually generated density maps that mimic the patterns of COVID-19 of varying case severity to develop algorithms with higher specificity. The results of this study also suggest the potential for this type of screening tool to discover infections that present critical risks for patients undergoing chemotherapy and radiation therapy, such as pneumonitis. Early identification of such infections would allow rapid intervention that is key for improving the outcome of these patients as their immune system is often compromised following their cancer treatment.
